# Defining and characterizing task-shifting medical devices

**DOI:** 10.1186/s12992-021-00684-6

**Published:** 2021-05-22

**Authors:** Amir Sabet Sarvestani, Marianna Coulentianos, Kathleen H. Sienko

**Affiliations:** 1 Design Science, University of Michigan, Ann Arbor, USA; 2 Department of Mechanical Engineering, University of Michigan, Ann Arbor, USA

**Keywords:** Task shifting, Medical devices, Low-income countries, Device characteristics, Task-shifting medical device, Ease of use, Healthcare worker, Health technology, Requirements

## Abstract

**Background:**

Task shifting could help address limited human resources available for the delivery of quality health care services in low-resource settings. However, the role of medical devices in supporting task shifting is not fully understood. This study aimed to 1) define “task-shifting medical devices” and 2) identify product characteristics to guide the design and development of task-shifting medical devices.

A three-part survey questionnaire comprising open-ended, rank-ordering, and multiple-choice questions was disseminated to healthcare professionals worldwide. The survey included questions to capture stakeholders’ general understanding of and preferences for task shifting in medicine and public health, and questions to define task-shifting medical devices and identify desirable product characteristics of task-shifting medical devices.

**Results:**

Task-shifting medical devices were defined by respondents as “devices that can be used by a less specialized health worker”. Aside from safe and effective, both essential characteristics for medical devices, easy to use was the most cited product characteristic for a task-shifting medical device. Responses also emphasized the importance of task-shifting medical devices to enable local agency, such as peer-to-peer training and local maintenance. Several additional frequently mentioned attributes included low cost, contextually appropriate, maintainable, capable of using an alternative power source, easy to understand, easy to learn, reusable, and easy to manage throughout its use cycle.

**Conclusion:**

This study defines and characterizes task-shifting medical devices based on healthcare professionals’ responses. Ease of use was identified as the most important characteristic that defines a task-shifting medical device, alongside safe and effective, and was strongly associated with enabling peer-to-peer training and maintainability. The findings from this study can be used to inform technology product profiles for medical devices used by lower-level cadres of healthcare workers in low-resource settings.

**Supplementary Information:**

The online version contains supplementary material available at 10.1186/s12992-021-00684-6.

## Background

Low-income countries (LICs) bear the highest burden of disease and are negatively affected by the lack of trained healthcare providers [1–3]. For example, sub-Saharan Africa comprised 11% of the world’s population in 2007 and bore 24% of the global disease burden with only 3% of the global health workforce [3]. Task shifting (TS) has been suggested, and is practiced, as a potential solution to address the limited available human resources for delivery of healthcare services in LICs [2, 3]. TS is a process whereby specific tasks are moved, where appropriate, to less-specialized health workers [1, 2, 4]. In recent years extensive systematic approaches have been developed to promote the shift of many basic healthcare delivery tasks for maternal and newborn health [2, 5–8] and HIV/AIDS [6, 9–12]. Prior studies suggest that the use of TS or task sharing for urgent and widely needed tasks may increase access to and availability of maternal and reproductive health services without compromising performance or patient outcomes, and may lead to cost effective practices [10, 13]. There are, however, potential barriers to effective implementation, such as possible adverse effects on patient safety, poor clinical support and supervision, inadequate training, and the lowering of standards of care [4, 10, 11, 14–16].

While TS has gained attention as a potential solution to address the limited health workforce in LICs, the role of medical devices for supporting TS is underexplored [4–6]. Examples of medical devices used in TS, such as PrePex and Shang Ring to assist with clinical male circumcision and Uniject for vaccine and drug delivery [6, 9, 12, 17], are rare. General target product profiles for TS medical devices do not exist, therefore, little is known about the typical characteristics needed to design and develop TS medical devices. The goals of this study were to 1) define TS medical devices, and 2) identify common product characteristics necessary for the development and implementation of TS medical devices, from the point of view of healthcare stakeholders from low-resource settings.

## Methods

A multi-part survey questionnaire comprising open-ended, rank-ordering, and multiple-choice questions was developed and administered using Qualtrics survey software (see survey questionnaire in Additional file 1). Section 1 included open-ended questions to capture stakeholders’ general understanding of and preferences for TS in medicine and public health. Section 2 included open-ended, rank-ordering, and multiple-choice questions to define TS medical devices and identify priority design characteristics. Open-ended questions were intentionally positioned in the first two sections of the survey to elicit unbiased responses, followed by closed-ended questions. Respondents could not revisit a question or section after completion, and could only view one question at a time.

Broad stakeholder participation was sought through online professional communities with members involved in healthcare delivery and technology development in LICs, e.g., LinkedIn’s Global Public Health. The survey questionnaire was also distributed to members of global networks in healthcare, e.g. the World Health Organization’s (WHO) Medical Devices Unit email list, and to health professionals and practitioners in Ethiopia, Ghana, and Uganda who had prior engagement with the research team. The survey was available online between June 15 and August 30, 2014, for public access.

An iterative inductive coding process was used to develop a thematic codebook based on the open-ended responses. The thematic analysis process involved identifying, reviewing, and naming emergent themes in the data. Medical device literature was consulted when refining a subset of theme names (e.g., safe, easy to use, low-cost). Multiple study team members trained in qualitative methods contributed to the identification, review, and naming of the themes. After multiple iterations of reviewing, combining, and naming, a final list of themes was established. One study team member applied the final codebook to the data and reviewed a subset of the codes with a second team member.

## Results

**Fig. 1:**
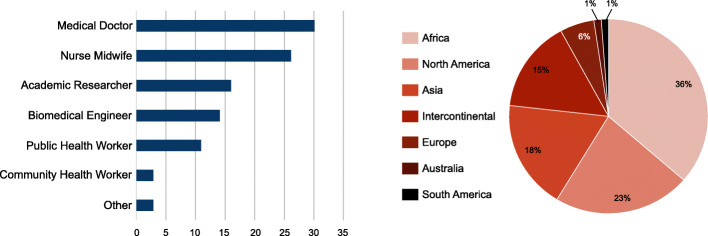
Respondent characteristics. Left: participant job titles. Right: participant location

Of the 350 respondents who initiated the survey response process, 107 respondents completed the survey, yielding a 30.6% completion rate. Three of the 107 respondents were removed for failure to complete the questionnaire properly, for a final total of 104 responses. Respondents were grouped into categories based on professional position and responding geography (see Fig. 1).

### Section 1

A total of 83 (80%) respondents had heard of, or knew about, TS. When asked to provide a definition of TS (Q1.1), of those that did provide a definition, 63 (76%) respondents’ definitions aligned with the WHO’s definition of TS: “Specific tasks are moved, where appropriate, from highly qualified health workers to health workers with shorter training and fewer qualifications in order to make more efficient use of the available human resources for health” [18]. Although most responses aligned with the WHO definition of TS, 9 (11%) respondents expressed that tasks do not necessarily have to be shifted down, instead, they should be shared. When asked to list conditions and prerequisites for TS (Q1.4), 27 (33%) respondents cited high demand and urgent need when few care providers are available. Furthermore, 32 (39%) respondents listed the need for continuous supervision and support as a prerequisite for TS (Q1.4).

### Section 2

Definitions of a TS medical device given by 49 (50%) respondents were categorized as: “the device can be used by a less specialized health worker”. Of these responses, 31 employed language such as “can use”, “enables”, “allows”. A subset of 24 (23%) respondents did not provide a definition.

When asked to provide design characteristics for a TS medical device (Q2.2), 71 responses were coded as “easy to use” (including “simple” (9 counts); “user friendly” (8 counts); “simple procedure” (7 counts); “simple to use” (5 counts); and “less manpower needed” (1 count)). Based on the data, separate codes for “easy to understand” and “easy to learn” were developed in addition to the “easy to use” code due to distinct differences among the responses. For example, “easy to understand” responses focused on the level of ease with which one could comprehend the purpose of the device or intuit the user interface without necessarily using the device (i.e., self-explanatory), as opposed to the level of ease with which one could physically operate the device, captured by the “easy to use” code. “Easy to learn” on the other hand, captured the ease with which one could learn to use the device during a pre-use/training phase, and respondents provided specific examples of preferred training approaches based on their prior experiences, noting the importance of efficient and adequate training programs (e.g., learning facilitated through formal training sessions organized by manufacturers or through peer-to-peer training).

The most cited characteristics are reported in Table 1.

**Table 1:** Most cited product characteristics, for a TS medical device

Counts	Characteristic	Example responses
64	Easy to use	• “Easy to use” • “Simple”; “simple procedure in operation”; “simple to use” • “User friendly”
49	Safe	• “Safe”; “error proof”
30	Low-cost	• “Cheap, low-cost” • “Cost efficiency”
29	Contextually appropriate and acceptable	• “Acceptable to staff and patients” • “Able to be discarded without damage to local environment” • “Have instructions in local language” • “Similarity to other devices used in that environment” • “Locally made”; “locally serviceable”; “locally available material and fits into local system”
27	Effective	• “Improves outcome consistently and safely” • “Accurate” • “Useful, dependable”
26	Maintainable	• “Easy to maintain / clean”
25	Uses an alternative power source	• “Renewable source of energy” • “Battery operated” • “Should be powered both mechanically and manually”
23	Easy to understand	• “Intuitive to operate (does not require detailed instructions to learn to use it)” • “Easy to understand for everyone”; “widely accessible”; “self-explanatory” • “Have some form of instructions on how to use them (labels, stickers, engravings, etc.)” • “Clear and simple instructions for use”
21	Easy to learn	• “Easy to learn how to use (training is less than a day)” • “Easy to teach on a peer-to-peer basis” • “Adequate training of users and retraining” • “Minimal instruction needed (if any)”
17	Easy to manage life-cycle	• “If reusable, device is easy to clean or prepare for next patient” • “Should not require complex assembling” • “Easy to dispose of”
17	Portable	• “Mobile” • “Easy to transport”

When asked to describe characteristics for an easy-to-use medical device (Q2.3), respondents provided similar characteristics as those shown in Table 1. However, the characteristics’ frequencies changed. When considering total counts of characteristics across Q2.1, Q2.2, and Q2.3 (reported in Table 2), two new characteristics emerged as salient: “uses advanced technology” (34 total counts, including “uses digital technologies”; “uses automation”; “manages data”; and “provides privacy protection”) and “simple design” (30 total counts including “few parts”, “few features”, “simple mechanism”).

**Table 2:** Compiled codebook of characteristics and counts across questions Q2.1, 2.2, 2.3

Characteristic	Total counts
Total number of characteristics coded	883
Ease of use-related characteristics	326
Easy to use	133
Easy to understand	79
Easy to learn	58
Easy to manage life-cycle	39
Easy to understand results	13
Other	4
Safe	91
Contextually appropriate, acceptable	60
Low-cost	49
Effective	46
Maintainable	45
Don’t know, unclear, no answer	41
Uses an alternative power source	37
Uses advanced technology	34
Portable	30
Simple design	30
Available, accessible	22
Ergonomic	20
Durable	15
Reusable	15
Disposable	12
Fast operation, reduces work burden	10

Lastly, in the multiple-choice question about the characteristics that make a medical device easy to use (Q2.7), respondents perceived a device to be easy to use if its operation could be “taught on a peer-to-peer basis” (90% of respondents selected this choice) and if it could be “maintained and repaired locally” (80%). In addition, the following characteristics were all selected by more than 50% of respondents: “easily cleaned by accessible and locally available cleaning products”; “culturally appropriate”; “effective immediately”; and “portable”. Notably, the characteristics “simple design” (30 counts), “no need to interpret results” (18 counts), and “error proof” (e.g., hard to use wrong) (15 counts) were highly recurrent in the open-ended question but were not listed in the list of 20 characteristics provided as part of the survey.

## Discussion

Overall, the respondents had a good understanding of the meaning of TS. Responses aligned with the definition of TS given by the WHO and with the WHO’s recommendations to extend TS where access to healthcare providers is constrained [18].

One of the few existing definitions of TS medical devices in the literature was provided by Jaroslawski and Saberwal (2013) who proposed that TS medical devices “should be explicitly designed to facilitate TS to lower cadres of workers” [19]. In their study, respondents defined a task-shifting medical device as a device that *enables* lower cadres to perform a procedure that was not typically part of their professional scope of work; hence, participants may not have assumed the complete shift of the task from higher- to lower-level cadres of workers. In practice, a device that is enabling could support lower-level cadres of workers to solely perform the task, and potentially also be used by higher-level cadres of workers in a task-sharing scenario. Prime et al. (2017) emphasized the simplifying role of TS medical devices: “In most cases, ‘TS’ is supported by a new protocol or product, which makes the previously complex task easier and safer” [20], which is consistent with the characteristics that emerged from this study.

The two design characteristics of a TS medical device that were cited most often by respondents were “easy to use” and “safe”. Safety is a primary requirement in medical device design [21], but oftentimes an implicit requirement, which may have ultimately affected the final counts, i.e., "safe" may have been an assumed characteristic resulting in the "easy to use" characteristic garnering the most counts.

In general, responses had a high occurrence of “intuitiveness” and “easy to learn” characteristics. The term “intuitive” in design is poorly understood [22], although it is present in user requirements, notably in medical device design requirements (e.g., “intuitive sample handling” [25]). Blackler et al. (2010) defined intuition as “cognitive processing that is often non-conscious and utilizes stored experiential knowledge”. Attributes of intuitive design have been defined for HCI applications [23], but limited research has explored the attributes of intuitive design with respect to medical devices.

The results from the multiple-choice question about what characteristics make a medical device easy to use were consistent with the responses from the open-ended questions Q2.2 and Q2.3, and they also demonstrated the importance of enabling local agency, such as enabling local users to train their peers and local technicians to maintain the devices. Devices that support peer-to-peer training and local maintenance would allow designers to potentially address some of the major issues that TS medical devices might face due to the remoteness of their intended use environment. Another means of promoting local agency is the use of locally available materials, as illustrated in multiple device examples in Howitt et al. (2012) [24]. Furthermore, the ease of understanding and ease of learning related responses gathered through this study were well aligned with general recommendations for [24] and research findings about operation and service manuals. McConnell (1995) suggests that further research is needed to determine what makes an instruction manual “user friendly” after showing that nurses in tertiary care hospitals in the midwestern US learn about medical devices mainly through trial and error and through reading the user instruction manual because of inadequate time to learn from suppliers’ demonstrations [25]. Furthermore, Wyatt (2008), and Ndlovu and Sibanda (2014) described the need for pictorial instruction manuals of medical devices to be used in low-resource settings to increase ease of understanding [26, 27]. Taken together, the multiple ease of use related characteristics identified in this study have implications for the safety and efficacy of TS medical devices, and regulatory bodies require medical device manufacturers to conduct usability testing to prevent patient injuries and death due to use errors [23].

The following eight main design characteristics for TS medical devices that emerged from the responses corresponded to characteristics of medical devices for resource-constrained, remote areas: “contextually appropriate”, “low-cost”, “effective”, “maintainable”, “uses an alternative power source”, “uses advanced technology”, “portable”, and “simple design”. Designing “contextually appropriate” medical devices was the focus of a paper by Aranda Jan et al. (2016); multiple dimensions of context that affect the successful implementation of medical devices in LICs, including infrastructural, socio-cultural, geographical and environmental, among others, were defined [28]. Howitt et al. (2012) also described important design characteristics for global health technologies similar to those uncovered in this study including maintainable, reliable power supply, portable, and low cost [24]. Malkin (2007) reported that the most common causes of failure among repaired medical equipment in low-resource settings were power supplies, user error, and failure to complete preventative maintenance [29].

Respondents were divided on the question of reusable versus disposable devices. Howitt et al. (2012) also discussed the tradeoffs of disposable devices, which are less environmentally friendly and have supply issues, and reusable devices, which need to be serviceable [24].

The seemingly opposing TS medical device characteristics that emerged as desirable in this study – “simple” devices with “few parts” and devices that “use advanced technology” – can be interpreted as aiming to achieve similar outcomes with respect to ease of use (as well as ease of learning to use and ease of understanding); although, it is recognized that some particularly simple health technologies, such as a scalpel, require considerable skill and training to use effectively in certain scenarios. Designers are therefore confronted with an oftentimes challenging trade-off: designing a device that automates a task thereby requiring minimal specialized user expertise, which oftentimes necessitates a more complex device, versus designing a simple device with a limited number of parts to minimize the complexity involved in maintaining the device, which oftentimes necessitates more specialized training.

The study’s outcomes rely on an online survey questionnaire that presents several limitations. For example, the background information provided by respondents was not validated and could have led to inaccurate demographic data, and the respondents could have searched for information prior to answering survey questions, which would have skewed results. A subset of respondents (22, 20%) acknowledged having little experience with TS, which may have negatively altered the aggregate responses related to the definition and characteristics of TS medical devices. On the other hand, biomedical engineers may have been more knowledgeable about general medical device characteristics, which may have contributed to the consistent responses that they provided as a group (e.g., 14 out of 16 biomedical engineers provided the response "used by a lesser trained worker" when defining TS medical devices). In addition, only including English-speaking respondents who worked in LICs undoubtedly limited the range of responses. Some stakeholder groups had very few respondents: Community health workers and Others had only three respondents each.

This study has implications for medical device design practice, especially concerning the design of devices for use in LICs. As TS initiatives increase, introducing TS medical devices could help alleviate some of the need for human support in TS, both reported by respondents and cited in the WHO recommendations [18], by lessening the stress felt by lesser trained healthcare workers engaging in medical procedures outside of the scope of their titles [30]. This study provides a better understanding of the potential role of medical devices in TS initiatives and of characteristics specific to TS medical devices. The findings from this study could further be used to inform technology product profiles for medical devices used by lower-level cadres of healthcare workers in low-resource settings.

Future work could involve investigating the potential tradeoffs between developing a TS medical device to enable health providers to perform a task versus training them to perform it without the device. While the necessity for TS must be confirmed on a case-by-case basis, it is important to understand at what point it is reasonable to spend time and financial resources to shift a task via adoption of a device versus additional training of healthcare providers.

## Conclusion

This work investigated the definition and characteristics of TS medical devices as expressed by stakeholders involved in health delivery, directly or indirectly, in LICs. TS medical devices were defined by respondents as “devices that can be used by a less specialized health worker,” a definition that could potentially support task sharing in addition to TS.

Elicited characteristics of TS medical devices aligned with general characteristics recommended for medical devices for use in LICs, where TS occurs most often. Alongside traditional characteristics for medical devices such as “safe” and “easy to use”, characteristics for TS medical devices included: enabling local agency, such as peer-to-peer training and local maintenance; contextually appropriate; capable of using an alternative power source; easy to understand; easy to learn; and easy to manage throughout its use cycle. The findings from this study can be used to inform technology product profiles for medical devices used by lower-level cadres of healthcare workers in low-resource settings.

## Supplementary Information

**Additional file 1.** Detailed survey questionnaire. An additional file providing the detailed survey questions used in the study is provided.

## Data Availability

The datasets used and analyzed in the current study are available from the corresponding author on reasonable request.

## Abbreviations

LICs: Low-Income Countries
TS: Task Shifting
WHO: World Health Organization
