# Supplementary material for: Defining and characterizing task-shifting medical devices
Source: Global Health. 2021 May 22;17:60. doi: 10.1186/s12992-021-00684-6 (PMC8140413; doi:10.1186/s12992-021-00684-6)
Supplement: Supplementary file 1 — Additional file 1. Detailed survey questionnaire. An additional file providing the detailed survey questions used in the study is provided. [file 12992_2021_684_MOESM1_ESM.pdf]

Appendix 1: Detailed survey questionnaire

Section 1: General Task Shifting principles

Q1.1. Have you heard of task shifting in healthcare delivery? If so, what does task shifting mean to you in the context of your work (in healthcare delivery)?

Q1.2. Considering task shifting means “when feasible, healthcare tasks are shifted from higher trained health workers to less highly trained health workers in order to maximize the efficient use of health workforce”, do you agree with this definition? If not, please explain why.

Q1.3. Considering the definition of task shifting, list a few examples (procedures, devices, etc.) from your field that you believe can be considered as task shifting.

Q1.4. Under what conditions and pre-requisites do you think task shifting can happen? The definition used for question 2 above was based on the World Health Organization’s (WHO) statement.

Section 2: Definition and characteristics elicitation for task-shifting medical devices

Q2.1. What does it mean for a medical device to be task shifting?

Q2.2. What design characteristics make a medical device task shifting?

Q2.3. What design characteristics make a medical device easy to use?

Q2.4. What are some examples of medical devices that can be considered as task shifting?

Q2.5. Rank-order characteristics that make a medical device task shifting.

Characteristics include:

a. The device is easy to use.

b. The device is widely available.

c. The device is widely accessible (it can be delivered to users per their request).

d. The device is low-cost (inexpensive).

e. The device's operation is easy-to-learn (in less than a day).

f. There is a policy in place for a device to become task shifting.

Q2.6. What other characteristics would make a medical device task shifting?

Q2.7. Would you consider a device easy to use, if it has any of the following characteristics:

a. If its effective operation can be learned within three days.

b. If its effective operation can be learned in less than a day.

c. If its operation can be taught on peer-to-peer basis.

d. If it is inexpensive (low-cost) compared to current practice.

e. If is has an extensive operational manual written in the local language.

f. If it has a brief operational manual in the local language.

g. If it does not have an operational manual (no need for it).

h. If it is portable (i.e., an average person can move/transport it without requiring assistance).

i. If it reduces the current number of procedural steps for a given procedure.

j. If it is easily cleaned by accessible or locally available cleaning products.

k. If it is single use (disposable after it is used once).

l. If it does not use electricity as its power source (powered mechanically).

m. If it is maintainable by local technicians.

n. If it is made from locally available materials.

o. If it is one size-fits-all.

p. If it is available in different sizes.

q. If it is culturally appropriate.

r. If it is safe for the intended patient.

s. If it is effective immediately.

t. If it is widely available.

Q2.8. “Design characteristics” are tangible or intangible features that defined a medical device such as: “device should be powered by mechanical source.” What other design characteristics, if any, make a medical device easy to use?
